# Correlation between time in range and serum uric acid in Chinese patients with type-2 diabetes: an observational cross-sectional study

**DOI:** 10.1186/s13098-024-01313-z

**Published:** 2024-03-21

**Authors:** Yan Liu, Xiaoren Peng, Chunjian Qiu, Jiaqing Shao

**Affiliations:** 1 Endocrinology Department, Jinling Hospital, Affiliated Hospital of Medical School, Nanjing University, 305 Zhongshan Dong Lu, Xuanwu District, Nanjing, 210000 Jingsu Province China; 2 Faculty of Clinical Medicine, Nanjing Medical University, 101 Longmian Avenue, Jiangning District, Nanjing, 210000 Jiangsu Province China

**Keywords:** Type-2 diabetes mellitus, Time in range, Hemoglobin A1C, Serum uric acid, Continuous glucose monitoring

## Abstract

**Background:**

At present, the relationship between serum uric acid and blood glucose is controversial, and even opposite conclusions have been reached. We aimed to investigate the relationship between time in range and serum uric acid and estimate the influence of serum uric acid on blood glucose fluctuations in Chinese patients with type-2 diabetes mellitus (T2DM).

**Methods:**

A total of 458 hospitalized patients with T2DM were selected. According to the SUA level, patients were divided into four groups by quartile: Q1 (≤ 254.5 µmol/L), Q2 (254.5–306.0 µmol/L), Q3 (306.0–385.5 µmol/L) and Q4 (> 385.5 µmol/L). The differences in general data, TIR and other clinical indicators between the four groups were assessed. Multifactor regression was used to analyze the relationship between subgroups of SUA and TIR, TBR, TAR, MAGE, SD, ADRR, MODD and M value. Curve fitting was used to analyze the association between TIR and SUA and to identify the inflection point.

**Results:**

TIR showed an overall increasing trend with increasing SUA, while HbA1c, TAR, MAGE, SD, ADRR, MODD and M value showed an overall decreasing trend with increasing SUA. Multivariate regression analysis showed that, compared with Q1, there was no correlation between SUA and TIR, TAR, ADRR, SD, or MODD in all models of Q2. In the Q3 and Q4 groups, SUA was correlated with SD, MODD, and MAGE in all models. In the Q4 group, SUA was correlated with TIR, TAR, ADRR, and the M value in all models. When SUA > 306 µmol/L (Q3 and Q4), TIR and SUA have a curve-like relationship, and the inflection point of the fitted curve was SUA = 460 mmol/L. Before the inflection point, β was 0.1, indicating that when SUA increases by 10 mmol/L, the corresponding TIR increases by 1%. After the inflection point, there was no significant difference in the correlation between TIR and SUA (P > 0.05).

**Conclusions:**

There is a close relationship between TIR and SUA in T2DM patients, it is speculated that SUA in a certain range had a positive protective effect on blood glucose control.

## Background

In recent years, an increasing amount of research has focused on the relationship between serum uric acid and blood glucose metabolism [1, 2]. Many studies have shown that hyperuricemia can impair islet function and increase insulin resistance, which is an independent risk factor for the occurrence and development of diabetes. A Swedish study showed that humans whose SUA reached a certain range over 14 years were six times more likely to have diabetes than those with the lowest SUA level [3]. Similarly, a 12 year follow-up analysis in the United Kingdom demonstrated that patients with the highest SUA levels had a 1.5-fold increased incidence of T2DM compared with the incidence among those with the lowest SUA levels [4]. A 3.5 year follow-up study in China observed similar results: Patients with greater baseline SUA levels had a 2.71-fold incidence of diabetes as those with lower baseline SUA levels [5]. A 5 year follow-up study in Israel discovered that a 1 mg/dL increase in SUA levels was related to a 1.14-fold increase in diabetes risk [6]. In a Finnish diabetes prevention study, humans with impaired glucose tolerance were twice as likely to develop T2DM among those with higher baseline SUA levels than those with lower baseline SUA levels [7]. The results of a cohort prospective study also showed that the baseline SUA level was an independent and strong predictor of diabetes development [6].

However, there are still many studies showing that hyperglycemia is associated with a low SUA level, especially in patients with diabetes. Cross-sectional studies have shown that SUA levels are low in patients with diabetes [8]. A prospective study of 10,000 humans reported that SUA levels were significantly greater in prediabetic patients than in nondiabetic patients but decreased among those who had diabetes [9]. Studies in Asian Indians [8] have shown that SUA levels increase significantly in patients with impaired glucose tolerance (IGT) and then decrease significantly as patients develop diabetes. The same report was found in a study of Caucasians, and more notably, uric acid levels tended to increase with blood glucose values until fasting blood glucose (FBG) was < 7.0 mmol/L in men and < 9.0 mmol/L in women. After crossing the inflection point, the SUA level decreased significantly with the blood glucose level [10]. At present, research mainly focuses on the correlation between hyperuricemia and the onset and progression of diabetes. The traditional view is that hyperuricemia is related to insulin resistance and is one of the risk factors for the development of diabetes. However, both domestic and foreign literature [11, 12] suggest that the higher level of SUA, the better the function of pancreatic β-cell. There are also research showing that SUA is negatively correlated with glycated hemoglobin. All the above suggests that SUA has a protective effect on pancreatic βcells [13] and can improve blood glucose control. In order to study the relationship between SUA and blood glucose control levels, we use the TIR measured by continuous glucose monitoring (CGM) as an indicator to evaluate the level of blood glucose control, and analyze the correlation between SUA and blood glucose control in patients with T2DM.

At present, the relationship between SUA and blood glucose is controversial, and even opposite conclusions have been reached. In order to better discover the relationship between SUA and blood glucose, TIR measured by means of a dynamic blood glucose monitor (CGM) was used as the vital indicator to evaluate blood glucose levels, and the correlation between SUA and blood glucose in T2DM patients was analyzed.

## Methods

### Study population

From January 2017 to December 2020, a total of 458 adult patients with T2DM were admitted to the Endocrinology Department of Nanjing Jinling Hospital, all of whom were confirmed to have T2DM according to the 1999 WHO diagnostic criteria. All patients with T2DM underwent treatment with oral medication and/or subcutaneous insulin. The exclusion criteria included (1) patients with type-1 or other types of diabetes; (2) patients who had acute complications of diabetes or acute stress, such as severe infection, trauma, surgery, severe respiratory disease, malignant disease, severe cardiovascular or cerebrovascular diseases, or pregnancy; (3) patients with hepatic or gallbladder diseases; (4) patients who had taken drugs affecting blood uric acid levels, such as diuretics, allopurinol, and benzbromarone febuxostat, within the last 3 months; and (5) patients who had been taking either narcotic or psychotropic drugs or both and patients with a recent history of alcoholism. The study was approved by the local ethics committee.

### Clinical and biochemical measurements

Clinical information and physical examination data, such as age, sex, diabetes duration, systolic blood pressure (SBP), diastolic blood pressure (DBP), height, weight and smoking history were collected through the medical records system. Body mass index (BMI) was calculated. Biochemical information, such as blood tests, was recorded after overnight fasting. Serum uric acid (SUA), hemoglobin A1C (HbA1c), fasting blood glucose (FPG), blood urea nitrogen (BUN), triglycerides (TGs), total cholesterol (TC), low-density lipoprotein (LDL), high-density lipoprotein (HDL), and serum creatinine (SCr) were detected.

### CGM parameters

The continuous glucose detection system from MiniMed Company and Meiqi Company was used in this study to continuously monitor glucose during a 72 h period, and patients’ capillary blood glucose was tested at least five times a day to update the monitoring process according to procedures. TIR was considered the value of the percentage of time during a 24 h period that glucose levels were within the range of 3.9–10 mmol/L. The date of time above range (TAR), time below range (TBR), mean amplitude of glycemic excursions(MAGE), standard deviation (SD), average daily risk range(ADRR), mean of daily differences (MODD) and M value were also collected.

### Statistical methods

The SPSS 22.0 software package was used for statistical analysis in this study. Continuous variables are expressed as the mean ± standard deviation when consistent with a normal distribution and as the median (upper and lower quartiles) when they were not normally distributed. Categorical data are expressed as percentages. Student’s t test was used to compare the samples conforming to a normal distribution. One-way analysis of variance (ANOVA) was used for comparisons among multiple samples, and the Kruskal‒Wallis test was used for comparisons among samples with abnormal distributions. The χ2-test was used for categorical variables. We used a smoothing function to fit the relationship between TIR and SUA. In addition, the piecewise linear regression model was used to test the influence of TIR on SUA by a smoothing function, and threshold effect analysis was performed to determine the inflection point. We also performed log-likelihood ratio tests for the single-line linear regression model and two-segment linear regression model. All analyses were performed using Empower (R) (www.empowerstats.com, X&Y Solutions, Inc.) (Boston, MA) and R (http://www.r-project.org). All tests were two-tailed, and the difference was considered statistically significant if the P value was less than 0.05.

## Results

### Baseline characteristics

According to SUA, 458 patients were divided into four groups by quartile, SUA Q1 (≤ 254.5 µmol/L), SUA Q2 (254.5–306.0) (µmol/L), SUA Q3 (306.0–385.5) (µmol/L) and SUA Q4 (> 385.5 µmol/L).

Male sex, smoking, weight, BMI, SCr, TG and TIR showed an overall increasing trend with the increase in SUA, while age, HDL, HbA1c, TAR, MAGE, SD, ADRR, MODD and M value showed an overall decreasing trend with the increase in SUA. The pairwise comparison between groups is shown in Table 1. There were no significant differences in SBP, DBP, TC, LDL, FBG, TBR and treatment or no treatment between subgroups.

**Table 1:** Characteristics of participants

Parameter	SUA Q1	SUA Q2	SUA Q3	SUA Q4	P
n	114	117	113	114	
Male, n (%)	46 (40.35)	72 (62.54)	88 (78.57)	96 (84.21)	< 0.001
Age (year)	58.72 ± 10.75	56.15 ± 11.59	52.21 ± 14.70^#^	51.27 ± 13.24^#*^	< 0.001
Weight (kg)	65.11 ± 10.55	68.14 ± 12.17	73.02 ± 12.90^#*^	76.77 ± 12.34^#*^	< 0.001
BMI (kg/m^2^)	24.05 ± 3.16	24.71 ± 3.66	25.35 ± 3.18^#^	26.32 ± 3.63^#*^	< 0.001
Diabetes duration (year)	9.87 ± 7.89	8.99 ± 6.65	6.97 ± 6.75^#^	7.60 ± 7.82	0.005
Smokingn (%)	19 (16.96)	33 (29.73)	43 (40.95)	40 (38.83)	< 0.001
SBP (mmHg)	134.18 ± 19.73	132.66 ± 15.57	135.29 ± 16.90	135.19 ± 18.73	0.547
DBP (mmHg)	78.58 ± 13.55	78.62 ± 9.20	80.95 ± 11.20	81.67 ± 13.31	0.261
SUA (µmol/L)	209.95 ± 33.66	281.43 ± 15.01^#^	341.90 ± 22.35^#*^	451.60 ± 65.34^#*∆^	< 0.001
BUN (mmol/L)	5.28 ± 1.32	5.74 ± 1.33	5.58 ± 1.47	6.92 ± 4.88^#*∆^	< 0.001
SCr (µmol/L)	49.50 ± 13.20	56.66 ± 16.25^#^	59.58 ± 15.30^#^	69.99 ± 19.44^#*∆^	< 0.001
TC (mmol/L)	4.28 (3.47, 5.01)	4.47 (3.73, 5.09)	4.43 (3.72, 5.08)	4.47 (3.89, 5.34)	0.359
TG (mmol/L)	1.17 (0.81, 1.70)	1.43 (0.96, 1.80)	1.67 (1.23, 2.77)^#*^	2.00 (1.17, 3.03)^#*^	< 0.001
HDL (mmol/L)	1.17 (1.00, 1.43)	1.10 (0.93, 1.31)^#^	0.99 (0.89, 1.14)^#*^	0.94 (0.84, 1.20)^#*^	< 0.001
LDL (mmol/L)	2.60(1.96, 3.24)	2.80 (2.12, 3.41)	2.72 (2.15, 3.32)	2.74 (2.13, 3.29)	0.629
FBG (mmol/L)	7.94 ± 2.96	7.54 ± 2.81	7.69 ± 2.53	7.41 ± 2.67	0.425
HbA1c (%)	9.51 ± 2.22	9.25 ± 2.01	8.96 ± 2.24	8.27 ± 1.86^#*^	< 0.001
TBR (%)	0.00 (0.00, 0.20)	0.00 (0.00, 0.00)	0.00 (0.00, 0.00)	0.00 (0.00, 0.00)	0.136
TIR (%)	63.35 (42.80, 83.07)	67.62 (37.93, 80.46)	67.27 (42.11, 83.69)	79.03 (59.89, 89.16)^#*∆^	< 0.001
TAR (%)	34.97 (16.59, 56.81)	31.92 (18.38, 62.07)	30.47 (15.59, 57.89)	20.97 (10.19, 39.77)^#*∆^	< 0.001
MAGE (mmol/L)	4.86 (3.94, 5.92)	4.85 (3.71, 6.11)	4.20 (3.28, 5.22)^#*^	3.58 (2.92, 4.82)^#*^	< 0.001
SD (mmol/L)	2.54 (2.06, 3.45)	2.65 (1.97, 3.18)	2.16 (1.74, 2.73)^#*^	1.95 (1.45, 2.61)^#*^	< 0.001
ADRR (mmol/L)	24.50 (17.96, 34.65)	24.37 (18.45, 36.93)	22.49 (15.20, 30.28)^#*^	18.39 (11.53, 27.01)^#*^	< 0.001
MODD (mmol/L)	2.29 (1.82, 3.23)	2.27 (1.61, 3.06)	1.94 (1.41, 2.69)^#^	1.74 (1.10, 2.31)^#*^	< 0.001
M value (mmol/L)	9.42 (4.23, 19.81)	8.36 (4.35, 18.42)	7.37 (3.12, 14.86)	4.29 (2.03, 11.13)^#*^	< 0.001
Treatment, n (%)					0.574
No treatment	95 (84.82)	93 (79.49)	88 (77.88)	94 (81.74)	
OHA	89 (79.46)	90 (76.92)	88 (77.88)	79 (68.70)	
Insulin	53 (47.32)	51 (43.59)	57 (50.44)	51 (44.35)	
OHA and insulin	48 (42.86)	41 (38.04)	52 (46.02)	44 (38.26)	

Normally distributed variables in the table are presented as the means ± SD, non-normally distributed values are presented as medians (25% and 75% interquartiles), and categorical variables are presented as frequencies (percentages). Student’s t-test was used for comparison of data with a normal distribution, Wilcoxon rank-sum test for those with abnormal distributions, and χ2 -test for categorical variables

*BMI* body mass index, *SBP* Systolic blood pressure, *DBP* Diastolic blood pressure, *SUA* serum uric acid, *BUN* blood urea nitrogen, *SCr* serum creatinine, *TC* total cholesterol, *TG* triglyceride, *HDL* high-density lipoprotein, *LDL* low-density lipoprotein, *FBG* fasting blood glucose, *HbA1c* Hemoglobin A1C, *TBR* time below range, *TIR* time in range, *TAR* time above range, *MAGE* mean amplitude of glycemic excursions, *SD* standard deviation, *ADRR* average daily risk range, *MODD* mean of daily differences, *OHA* Oral hypoglycemic agents

vs Q1 group, ^#^P < 0.05

vs Q2 group, ^*^P < 0.05

vs Q3 group, ^∆^P < 0.05

### Factors affecting TIR

Univariate regression analysis showed that SUA, sex, weight, diabetes duration, smoking, SCr and HbA1c were all influencing factors of TIR (Table 2).

**Table 2:** Association between TIR and other indicators

	β	(95%CI)	P value
Sex			
0	−	−	
1	9.16	(4.43, 13.89)	< 0.001
Age	− 0.13	(− 0.31, 0.04)	0.140
Weight	0.19	(0.01, 0.37)	0.036
BMI	0.09	(− 0.56, 0.74)	0.796
Diabetes duration	− 0.51	(− 0.82, − 0.21)	0.001
Smoking, n(%)	5.141	(0.036,10.246)	0.048
SBP	− 0.14	(− 0.26, − 0.01)	0.036
DBP	0.13	(− 0.06, 0.32)	0.179
SUA	0.05	(0.02, 0.07)	< 0.001
BUN	0.29	(− 0.53, 1.11)	0.491
SCr	0.24	(0.12, 0.37)	< 0.001
TC	− 1.65	(− 3.69, 0.39)	0.113
TG	− 0.77	(− 2.07, 0.53)	0.247
HDL	0.44	(− 3.06, 3.94)	0.804
LDL	− 2.12	(− 4.58, 0.34)	0.092
HbA1c	− 5.58	(− 6.54, − 4.62)	< 0.001
Treatment			
0	−	−	
1	− 1.37	(− 7.25, 4.50)	0.646

### The effect of sex on the relationship between TIR and SUA

A hierarchical interaction test was used to explore the effect of sex on the relationship between TIR and SUA (Table 3). Before adjusting for any factors, the relationship between TIR and SUA existed in male participants but not in female participants. After adjusting for other influencing factors, the relationship between TIR and SUA still existed in male patients in Model I (P < 0.05), but not in Model II (P > 0.05). In female patients, the relationship was not significant after adjusting for other factors (P > 0.05). After interaction analysis, it was found that sex did not affect the relationship between TIR and SUA, regardless of whether other factors were adjusted.

**Table 3:** Hierarchical interaction analysis of the impact of sex on the relationship between TIR and SUA

Model	Male			Female			P value for interaction
	β	(95%CI)	P value	β	(95%CI)	P value	
Non-adjusted	0.04	(0.01, 0.07)	0.008	0.03	(− 0.01, 0.08)	0.115	0.855
Model I	0.04	(0.01, 0.07)	0.007	0.04	(− 0.00, 0.08)	0.067	0.953
Model II	0.02	(− 0.00, 0.05)	0.079	0.01	(− 0.03, 0.05)	0.753	0.435

Model I: Adjusted age, Diabetes duration, DBP, SBP, Weight and BMI

Model II: Adjusted Model I, FBG, SUN, SCr, TC, TG, HDL, LDL and HbA1c

*β* Regression coefficient, *CI* Confidence interval, *SUA* serum uric acid, *DBP* Diastolic blood pressure, *SBP* Systolic blood pressure, *BMI* Body mass index, *FBG* fasting blood glucose, *BUN* blood urea nitrogen, *SCr* serum creatinine, *TC* total cholesterol, *TG* triglyceride, *HDL* high-density lipoprotein, *LDL* low-density lipoprotein, *HbA1c* Hemoglobin A1c

### The effect of smoking on the relationship between TIR and SUA

A hierarchical interaction test was used to explore the effect of smoking on the relationship between TIR and SUA (Table 4). Before adjusting for any factors, the association between TIR and SUA was present in patients without a history of smoking, but not in patients with a history of smoking. After adjusting for other influencing factors, the relationship between TIR and SUA still existed in patients without a history of smoking in Model I (P < 0.05), but not in Model II (P > 0.05). In patients with a history of smoking, the relationship was not significant after adjusting for other factors (P > 0.05). After interaction analysis, it was found that smoking history did not affect the relationship between TIR and SUA, regardless of whether other factors were adjusted.

**Table 4:** Hierarchical interaction analysis of the impact of smoking on the relationship between TIR and SUA

Model	No smoke			smoke			P value for interaction
	β	(95%CI)	P value	β	(95%CI)	P value	
Non-adjusted	0.05	(0.02, 0.08)	< 0.001	0.04	(− 0.00, 0.09)	0.080	0.795
Model I	0.04	(0.02, 0.07)	0.003	0.05	(− 0.00, 0.09)	0.054	0.994
Model II	0.01	(− 0.02, 0.03)	0.678	0.01	(− 0.03, 0.05)	0.475	0.697

Model I: Adjusted age, Diabetes duration, DBP, SBP, Weight and BMI

Model II: Adjusted Model I, FBG, SUN, SCr, TC, TG, HDL, LDL and HbA1c

*β* Regression coefficient, *CI* Confidence interval, *SUA* serum uric acid, *DBP* Diastolic blood pressure, *SBP* Systolic blood pressure, *BMI* Body mass index, *FBG* fasting blood glucose, *BUN* blood urea nitrogen, *SCr* serum creatinine, *TC* total cholesterol, *TG* triglyceride, *HDL* high-density lipoprotein, *LDL* low-density lipoprotein, *HbA1c* Hemoglobin A1c

According to the SUA level, patients were divided into four groups by quartile: Q1 (≤ 254.5 µmol/L), Q2 (254.5–306.0 µmol/L), Q3 (306.0–385.5 µmol/L) and Q4 (> 385.5 µmol/L).

Multivariate regression analysis showed that, compared with Q1, there was no correlation between SUA and TIR, TAR, ADRR, SD, MODD in all models of Q2. In the Q3 and Q4 groups, SUA was correlated with SD, MODD, and MAGE in all models. In the Q4 group, SUA was correlated with TIR, TAR, ADRR, and M value in all models (Table 5).

**Table 5:** Association between SUA and TIR and other indices of glycemic fluctuation after SUA quartile stratification

Exposure	Non-adjusted			Model I			Model II		
	β	(95%CI)	P value	β	(95%CI)	P value	β	(95%CI)	P value
TIR									
SUAQ1	−			−			−		
SUAQ2	− 0.71	(− 6.98, 5.57)	0.826	− 2.32	(− 8.67, 4.02)	0.474	− 1.10	(− 7.47, 5.27)	0.735
SUAQ3	1.44	(− 4.91, 7.78)	0.657	− 1.41	(− 8.13, 5.31)	0.682	1.49	(− 5.13, 8.12)	0.659
SUAQ4	11.71	(5.39, 18.03)	0.001	9.99	(3.16, 16.81)	0.004	11.84	(4.50, 19.19)	0.002
TBR									
SUAQ1	−			−			−		
SUAQ2	− 0.05	(− 0.64, 0.54)	0.871	0.11	(− 0.51, 0.73)	0.736	− 0.01	(− 0.61, 0.60)	0.981
SUAQ3	− 0.51	(− 1.10, 0.08)	0.092	− 0.22	(− 0.87, 0.44)	0.521	− 0.36	(− 0.99, 0.27)	0.261
SUAQ4	− 0.53	(− 1.12, 0.06)	0.082	− 0.21	(− 0.87, 0.46)	0.545	− 0.29	(− 0.99,0.41)	0.419
TAR									
SUAQ1	−			−			−		
SUAQ2	1.00	(− 5.35, 7.34)	0.758	2.48	(− 3.95, 8.92)	0.450	1.32	(− 5.11, 7.74)	0.688
SUAQ3	− 0.93	(− 7.35, 5.49)	0.777	1.89	(− 4.93, 8.70)	0.588	− 1.19	(− 7.87, 5.49)	0.727
SUAQ4	− 10.94	(− 17.33, − 4.56)	0.001	− 9.50	(− 16.42,− 2.58)	0.007	− 11.41	(− 18.82,− 4.00)	0.003
SD									
SUAQ1	−			−			−		
SUAQ2	− 0.13	(− 0.39, 0.13)	0.334	− 0.06	(− 0.33, 0.22)	0.691	− 0.14	(− 0.41, 0.13)	0.298
SUAQ3	− 0.53	(− 0.80, − 0.27)	< 0.001	− 0.42	(− 0.71, − 0.13)	0.004	− 0.48	(− 0.76, − 0.20)	0.001
SUAQ4	− 0.77	(− 1.04, − 0.50)	< 0.001	− 0.70	(− 0.99, − 0.41)	< 0.001	− 0.73	(− 1.04, − 0.42)	< 0.001
MODD									
SUAQ1	−			−			−		
SUAQ2	− 0.17	(− 0.47, 0.13)	0.257	− 0.11	(− 0.41, 0.20)	0.497	− 0.18	(− 0.48, 0.13)	0.252
SUAQ3	− 0.51	(− 0.81, − 0.22)	0.001	− 0.42	(− 0.74, − 0.11)	0.010	− 0.47	(− 0.78, − 0.16)	0.003
SUAQ4	− 0.72	(− 1.02, − 0.42)	< 0.001	− 0.65	(− 0.98, − 0.32)	0.001	− 0.65	(− 1.01, − 0.29)	0.004
ADRR									
SUAQ1	−			−			−		
SUAQ2	0.71	(− 2.70, 4.12)	0.684	1.93	(− 1.55, 5.41)	0.278	0.96	(− 2.52, 4.44)	0.590
SUAQ3	− 3.18	(− 6.59, 0.23)	0.069	− 1.18	(− 4.82, 2.47)	0.527	− 2.31	(− 5.89, 1.27)	0.207
SUAQ4	− 7.39	(− 10.82, − 3.95)	< 0.001	− 5.91	(− 9.66, − 2.16)	0.002	− 5.98	(− 10.05,− 1.91)	0.004
MAGE									
SUAQ1	–			–			–		
SUAQ2	− 0.03	(− 0.49, 0.43)	0.908	0.07	(− 0.40, 0.54)	0.763	− 0.10	(− 0.56, 0.37)	0.687
SUAQ3	− 0.96	(− 1.42, −0.49)	< 0.001	− 0.84	(− 1.34, − 0.34)	0.001	− 0.90	(− 1.38, − 0.41)	< 0.001
SUAQ4	− 1.22	(− 1.69, − 0.76)	< 0.001	− 1.15	(− 1.66, − 0.63)	< 0.001	− 1.18	(− 1.72, − 0.64)	< 0.001
M value									
SUAQ1	−			−			−		
SUAQ2	− 2.75	(− 8.06, 2.57)	0.312	− 1.60	(− 7.19, 3.98)	0.574	− 2.46	(− 7.97, 3.06)	0.383
SUAQ3	− 5.97	(− 11.34, − 0.61)	0.030	− 4.31	(− 10.21, 1.59)	0.153	− 4.97	(− 10.69, 0.75)	0.089
SUAQ4	− 7.91	(− 13.25, − 2.58)	0.004	− 6.67	(− 12.66, − 0.68)	0.030	− 6.75	(− 13.09, -0.41)	0.038

Model I: Adjusted age, sex, Diabetes duration, smoking, DBP, SBP, Weight and BMI

Model II: Adjusted Model I, FBG, SUN, SCr, TC, TG, HDL, LDL, HbA1c

*β* Regression coefficient, *CI* Confidence interval,*TBR* time below range, *TIR* time in range, *TAR* time above range, *SD* standard deviation, *ADRR* average daily risk range, *MODD* mean of daily differences, *MAGE* mean amplitude of glycemic excursions

### Smoothing function (SUA > 306 µmol/L) and the turning point

In this study, when SUA was at a low level (SUA < 306 µmol/L), there was no significant correlation between SUA and TIR and other indices of blood glucose fluctuation. When SUA was at a higher level (SUA > 306 µmol/L), SUA was correlated with TIR and other indices of blood glucose fluctuation.

**Fig. 1:**
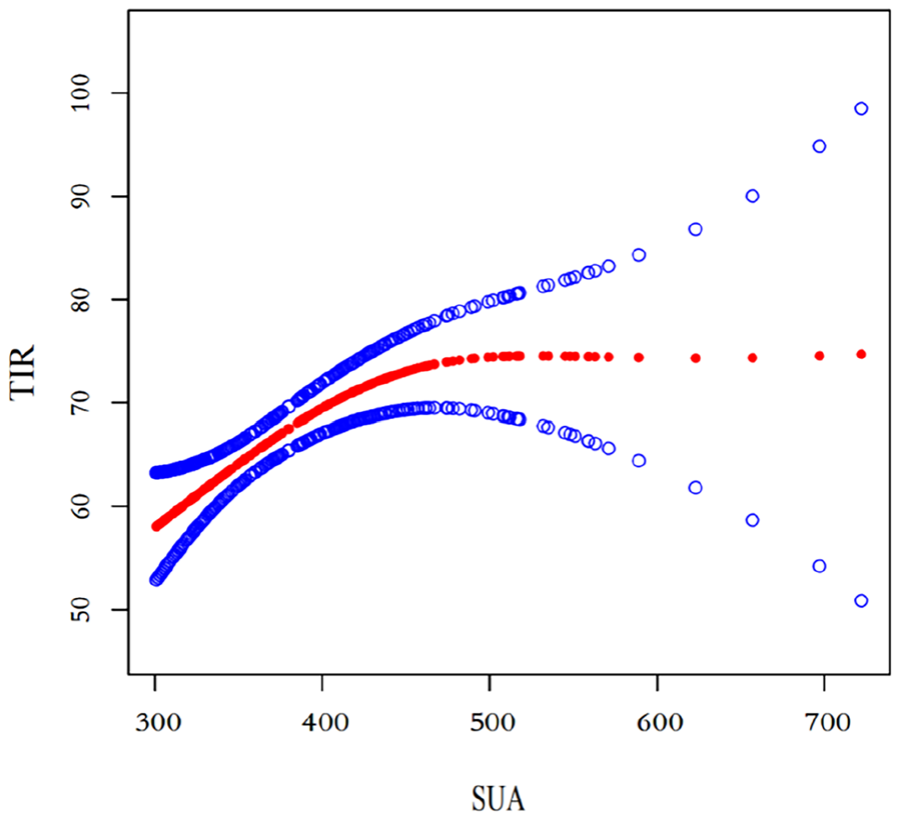
Smooth curve fitting diagram (SUA > 306 µmol/L)

Figure 1 is the fitting diagram of the smooth curve (SUA > 306 µmol/L). The abscissa is SUA, the ordinate is TIR, the solid line in the middle is the fitting line, and the dashed lines on both sides show the 95% CI. It can be seen from the figure that TIR and SUA have a curve-like relationship, and threshold and saturation effect analyses were conducted to accurately identify the inflection point (Table 6). The inflection point of the fitted curve was SUA = 460 mmol/L. Before the inflection point, β was 0.1, indicating that when SUA increases by 1 unit, the corresponding TIR increases by 0.1 units. After the inflection point, there was no significant difference in the correlation between TIR and SUA (P > 0.05).

**Table 6:** Saturation effect analyses were conducted to identify the inflection point

For exposure: SUA		
Outcome: TIR		
Inflection point (k)	460	
	β (− 1.55, 5.41)	P value
< K segment effect 1	0.1 (0.1, 0.2)	< 0.001
> K segment effect 2	− 0.0 (− 0.1, 0.1)	0.359
Log-likelihood ratio test		0.015

## Discussion

HbA1c is commonly used to evaluate the level of blood glucose control. However, HbA1c has limitations. The DCCT study found that HbA1c explained only 11% of diabetes complications, and 89% of diabetes problems no longer needed explanation, once speculated to be related to variability in blood sugar. Clinical studies have shown that the repeated fluctuation of the hyperglycemic environment causes more serious damage to the morphology and function of endothelial cells than continuous safe hyperglycemia [14], which is more likely to lead to microangioplasia and cardiovascular disease in T2DM patients. With the development of blood glucose monitoring technology, CGM can be applied to evaluate the blood glucose of patients. A new index, TIR, can directly reflect whether the blood glucose level reached the optimal level under various interventions. Lu [15] studied TIR assessed by CGM in 2215 patients with T2DM and carotid intima-media thickness (CIMT), a legitimate marker of subclinical atherosclerosis. The results showed that TIR in patients with abnormal thickening (≥ 1.0 mm) was significantly lower than that in patients with normal CIMT. For each 10% increase in TIR, the risk of abnormal CIMT was reduced by 6.4 percentage points, suggesting that TIR may play an additional predictive role in atherosclerosis progression. TIR is associated not only with macrovascular complications but also with microvascular complications of diabetes. A survey of 3262 patients with T2DM confirmed that the incidence and severity of diabetic retinopathy (DR) were negatively correlated with TIR but not with HbA1c [16]. Studies on TIR in diabetic patients have shown that TIR is significantly correlated with the incidence of retinopathy and microalbuminuria in T1DM patients. For each 10% TIR restriction, the risk of microalbuminuria increased by 40%, and the risk of DR increased by 64% [17]. Guo [18] analyzed the association between diabetic cardiovascular autonomic neuropathy (CAN) and TIR in a study including 349 T2DM patients and determined a reliable association between TIR and CAN independent of HbA1c. In conclusion, the clinical significance of TIR has been widely recognized. TIR was used as the main indicator to evaluate blood glucose levels in this study.

SUA is the product of purine metabolism and is an important component of cellular deoxyribonucleic acid (DNA). The uric acid concentration in humans is 3–10 times higher than that in other mammals [19]. According to evolutionary theory, the existence of reasonable uric acid in humans is conducive to evolutionary survival. However, due to changes in modern social lifestyle, uric acid can accumulate in the body as a result of excessive nutrition or nutritional imbalance, leading to metabolic disorders, which result in a series of medical issues. Hyperuricemia can lead to gout, chronic kidney disease, coronary heart disease, metabolic syndrome and other diseases. However, uric acid has clear and effective antioxidant and anti-inflammatory effects. Clinical hyperuricemia is occasionally a compensatory increase induced as a means of the body to combat against pathological stimuli or continual low-grade inflammation. To date, the results of uric acid in human diseases are controversial. Therefore, academic research on the physiological and pathological consequences of uric acid has been an important focus.

The results of the correlation analyses in this study revealed that weight, BMI, SCr and TG showed an overall increasing trend with the increase in SUA, and the variations were statistically significant (Table 1). According to the Third National Health and Nutrition Survey in the United States, the incidence of metabolic syndrome (MetS) increases drastically with the increase in serum uric acid [20], which is typically a group of conditions closely associated with lifestyle and characterized by obesity, hyperglycemia, fatty liver and dyslipidemia [21]. Intake of TG-rich meals will lead to hyperpurine synthesis and then increased SUA production. Moreover, the products of fat metabolism will inhibit the excretion of SUA. Conversely, the increase in SUA levels promotes lipid oxidation and peroxidation, leading to dyslipidemia [22].

Oxidative stress is an important factor that leads to insufficient insulin secretion and accelerates the progression of T2DM. It is possible that oxidative stress induced by reactive oxygen and nitrogen species is closely associated with β-cell dysfunction in the development of diabetes [23, 24]. The oxidative stress environment can cause insulin resistance, β-cell dysfunction, impaired glucose tolerance, and mitochondrial dysfunction, which may ultimately lead to the occurrence and progression of diabetes [25]. Basic studies have shown that uric acid can inhibit nitrification mediated via nitrite peroxide with the aid of scavenging peroxide, hydroxyl and oxygen free radicals; enhance the antioxidant levels of erythrocyte membrane lipids; and decrease oxidative stress in the body [26]. Some studies suggest that higher levels of SUA are associated with better β-cell function. There are various methods for clinically assessing β-cell function, and the arginine stimulation test can effectively evaluate the first-phase secretion function of β-cells [27, 28]. A Chinese study [29] on the correlation between blood uric acid levels and β-cell function in patients with T2DM, a multi-angle analysis of the data from the arginine stimulation test was conducted, leading to the conclusion that high levels of uric acid have a protective effect on β-cell function in T2DM patients.

In this study, it was found that TIR showed an overall increasing trend with the increase in SUA, and the differences among Q4 vs Q1, Q4 vs Q2, and Q4 vs Q3 were all statistically significant. In addition, TAR, MAGE, SD, ADRR, MODD, and M value showed an overall decreasing trend with the increase in SUA. It was suggested that the increase in SUA was related to the better control and stability of blood glucose in T2DM. Multiple regression analysis showed that no matter whether other factors were adjusted, the relationship between SUA and TIR persisted in Q3 and Q4 groups, while the correlation was not significant in Q1 and Q2 groups. The results suggested that the higher the concentration of SUA, the more obvious the correlation with TIR and other blood glucose control indexes. As seen from the smooth curve fitting diagram of TIR and SUA (Fig. 1), TIR and SUA have a curve-like relationship, and the log-likelihood ratio test shows that there is a significant nonlinear relationship between them (P > 0.05). The inflection point of the fitted curve was SUA = 460 mmol/L. Before the inflection point, β was 0.1, indicating that when SUA increases by 10 mmol/L, the corresponding TIR increases by 1%. This could be attributed to the fact that SUA is a major antioxidant substance in the blood and exhibits significant antioxidant effects. The antioxidant properties of SUA can help eliminate various substances, including singlet oxygen, peroxyl radicals, and hydroxyl radicals, thereby reducing metabolic inflammation, improving insulin resistance, and promoting insulin secretion. Finally, the antioxidant effect of SUA may have a protective effect on β cell function and a positive effect on blood glucose control in T2DM patients.

Additionally, in the real world, the positive effects of uric acid are getting more and more attention, and this shows up in other areas. The antioxidant effect of uric acid can manifest through its shielding impact on nerves [30, 31]. Llull et al. [32] discovered in patients with acute ischemic stroke that the use of uric acid blended with alteplase may reduce the ischemic area of cerebral infarction; consequently, it was speculated that uric acid had a neuroprotective effect. Ye et al. [33] studied 271 healthy subjects, 596 patients with slight cognitive impairment and 97 patients with Alzheimer’s ailment (AD), to assess the effect of uric acid on cognitive characteristics. The results confirmed that an excessive serum uric acid level should slow cognitive decline in sufferers with moderate cognitive impairment and in the AD subgroup, especially in female patients. This finding suggests that higher levels of uric acid have a protective effect against cognitive decline in nondementia patients. Uric acid protects the human body and can also affect immune function. For example, Ma Xiaojun [34] used uric acid to treat in vitro cultured mature mouse bone marrow-derived dendritic cells (BMDCs) and assessed immune characteristics. The in vitro-precipitated augmentation of BMDCs and uric acid promoted differentiation and maturation, instantly stimulating molecules on the surface and increasing the potential to stimulate T-cell proliferation and IL-12 secretion levels. The effect of uric acid was associated with its concentration.

In our study, there was no significant correlation between TIR and SUA after the inflection point. This may be due to the small sample size of patients with SUA levels greater than 460 and the potential damage to the body caused by excessively high SUA levels. Since there are few basic and clinical studies on the effects of different levels of blood uric acid on glycemic control, we are willing to continue to monitor this relationship in future studies.

## Conclusion

The results showed that TIR had an overall increasing trend with the increase in SUA. In a certain range, TIR and SUA have a curve-like relationship, and it is speculated that SUA had a positive protective effect on blood glucose control. We suggest that Chinese patients with T2DM can appropriately maintain SUA at a higher level within the normal range. However, because this was a cross-sectional study, further studies, especially prospective cohort studies and related physiological and pathological studies, are still needed to clarify the role of SUA levels on blood glucose states.

## Data Availability

The datasets generated during and/or analysed during the current study are available from the corresponding author on reasonable request.

## Abbreviations

T2DM: Type-2 diabetes mellitus
BMI: Body mass index
SBP: Systolic blood pressure
DBP: Diastolic blood pressure
SUA: Serum uric acid
BUN: Blood urea nitrogen
SCr: Serum creatinine
TC: Total cholesterol
TG: Riglyceride
HDL: High-density lipoprotein
LDL: Low-density lipoprotein
FBG: Fasting blood glucose
HbA1c: Hemoglobin A1C
TBR: Time below range
TIR: Time in range
TAR: Time above range
MAGE: Mean amplitude of glycemic excursions
SD: Standard deviation
ADRR: Average daily risk range
MODD: Mean of daily differences
OHA: Oral hypoglycemic agents
